# Supplementary material for: The interactome of intact mitochondria by cross-linking mass spectrometry provides evidence for coexisting respiratory supercomplexes
Source: Mol Cell Proteomics. 2017 Dec 8;17(2):216–32. doi: 10.1074/mcp.RA117.000470 (PMC5795388; doi:10.1074/mcp.RA117.000470)
Supplement: Supplemental Data [file supp_RA117.000470_133922_0_supp_23978_fzffwf.zip › spectra_annotation/mito_DR_spectra_annotation/425-1-7-1-17-1.pdf]

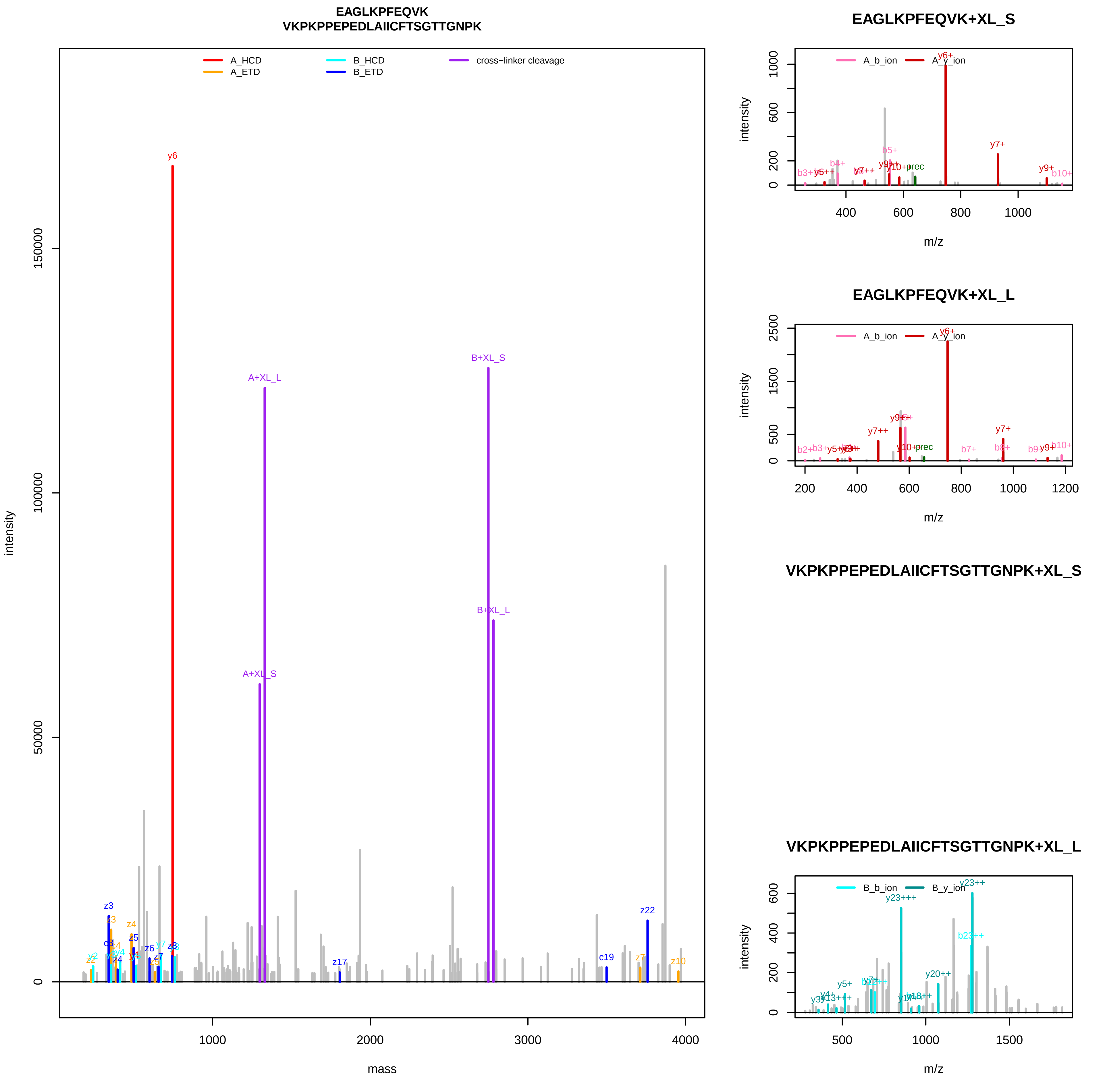
EAGLKPFEQVK
VKPKPPEPEDLAIICFTSGTTGNPK
A_HCD
A_ETD
B_HCD
B_ETD
cross−linker cleavage
y6
A+XL_L
A+XL_S
B+XL_S
B+XL_L
z3
z22
c19
z17
z10
intensity
mass
EAGLKPFEQVK+XL_S
A_b_ion
A_y_ion
y6+
y7+
y9+
b10+
b5+
b4+
b3+
y5++
y7++
y10++
prec
m/z
EAGLKPFEQVK+XL_L
b7+
b8+
b9+
b2+
y7++
VKPKPPEPEDLAIICFTSGTTGNPK+XL_S
VKPKPPEPEDLAIICFTSGTTGNPK+XL_L
B_b_ion
B_y_ion
y23++
y23+++
b23++
y20++
y5+
y4+
